# Refining Genetic Instruments for Dietary Intake Mendelian Randomization Using Phenome‐Wide Association Studies

**DOI:** 10.1002/gepi.70059

**Published:** 2026-10-01

**Authors:** Kristen J. Sutton, Julie E. Gervis, Moomal Jatoi, Liang‐Dar Hwang, Audrey E. Hendricks, Debashis Ghosh, Kenneth E. Westerman, Joanne B. Cole

**Affiliations:** ^1^ Department of Biomedical Informatics University of Colorado Anschutz Aurora Colorado USA; ^2^ Center for Genomic Medicine Massachusetts General Hospital Boston Massachusetts USA; ^3^ Metabolism Program Broad Institute of MIT and Harvard Cambridge Massachusetts USA; ^4^ Department of Medicine Harvard Medical School Boston Massachusetts USA; ^5^ Institute for Molecular Bioscience University of Queensland Brisbane Queensland Australia; ^6^ Department of Mathematical and Statistical Sciences University of Colorado Denver Denver Colorado USA; ^7^ Department of Biostatistics and Informatics University of Colorado Anschutz Aurora Colorado USA; ^8^ Clinical and Translational Epidemiology Unit, Department of Medicine Massachusetts General Hospital Boston Massachusetts USA

**Keywords:** causal inference, dietary intake, direct effect, functional variant, mendelian randomization

## Abstract

Most Mendelian randomization (MR) of dietary intake use the full set of genome‐wide significant (GWS) variants in the instrumental variable (IV), likely biasing causal estimates due to pleiotropy. To characterize the common methods to handle pleiotropy in dietary intake MR, we conducted a scoping review of the literature on dietary intake MR studies. We extracted information on IV construction, assessment of pleiotropy, and sensitivity analyzes revealing that only 20% of studies used an IV with functional plausibility. In the absence of functionally‐informed IVs, we tested if two‐sample MR using GWS variants filtered for pleiotropic associations through phenome‐wide association studies (PheWAS) could identify diet–health relationships supported by existing nutrition science, focusing on oily fish and alcohol intake, the latter of which has a functionally‐informed IV for comparison (rs1229984 in the *ADH1B* gene). To further explore this question, we performed multivariable MR and employed MR‐CAUSE. The numerous models consistently supported that oily fish reduced triglycerides. In contrast, GWS and PheWAS‐filtered IVs suggested that alcohol decreased alanine aminotransferase levels, whereas the functional IV (rs1229984) found the opposite expected relationship. Isolating the direct effect of dietary intake from GWS IV remains challenging. Future work should focus on identifying functional variants impacting dietary behavior.

## 1 Introduction

Dietary intake has profound effects on health. However, many relationships between foods and health outcomes remain controversial, and multiple robust associations from observational studies have failed in randomized controlled trials (Casas et al. 2005; Klein et al. 2011). Mendelian randomization (MR) is a form of instrumental variable analysis that has been likened to a randomized controlled trial by leveraging the random assignment of alleles at meiosis to estimate causal effects of an exposure, like dietary intake, on health outcomes. MR promises to be a cost‐effective intermediate tool to identify and clarify how foods affect health prior to undertaking long and expensive randomized trials.

In order for MR to accurately estimate causal effects, three main conditions must be satisfied: (1) relevance: the IV is associated with the exposure; (2) exchangeability: the IV is independent of confounders of the exposure‐outcome relationship; and (3) exclusion restriction: there are no paths from the IV to the outcome that do not go through the exposure (Sanderson et al. 2022).

In recent years, multiple genome‐wide association studies (GWAS) of food liking (May‐Wilson et al. 2022) and dietary intake (Cole et al. 2020; Meddens et al. 2021; Millard et al. 2018) have identified hundreds of genetic associations. For most associations the biological mechanism connecting the genetic variant with intake remains unknown. Furthermore, many diet‐associated loci are also associated with socioeconomic status, physical activity levels, and diseases, raising the possibility that these variants influence health outcomes through pathways independent of dietary intake (Cole et al. 2020). If alternative pathways exist, the exclusion restriction assumption underlying MR would be violated, potentially biasing the causal estimates.

Exposures with variants of known biological consequence allow more informed assessment of potential horizontal pleiotropy and, therefore, the plausibility of the exclusion restriction assumption. Variants with known biological mechanisms impacting dietary intake have been identified for dairy (lactase, *LCT*, rs4988235) (Enattah et al. 2002), alcohol (alcohol dehydrogenase, *ADH1B*, rs1229984; aldehyde dehydrogenase, *ALDH2*, rs671) (Thomasson et al. 1993), and caffeine (cytochrome P450 family 1 subfamily a polypeptide ½, *CYP1A1/2*, rs762551; aryl hydrocarbon receptor, *AHR*, rs6968865) (Sachse et al. 1999). Even though biologically informed variants tend to be more reliable and interpretable than GWS‐IVs, few are known, which greatly limits the use and necessitates less powerful single‐variant MR models.

Given the limitations of functional IVs, the recent availability of numerous and large dietary intake GWAS, and the ease of performing two‐sample MR analyzes with user‐friendly R packages and online portals, a surge of MR studies have occurred – often at the expense of the exclusion restriction assumption. Therefore, we first conducted a brief critical assessment of the dietary intake MR literature to identify common methods for building the IV and estimating the causal relationships. Second, we tested if, in the absence of known functionally informed IVs, we could reproduce diet‐health relationships supported by nutrition science with data‐driven approaches that use external GWAS data and PheWAS to filter the IV.

## 2 Methods

### 2.1 Scoping Review of Dietary Intake MR

The Medical Subject Headings (MeSH) terms for “Diet, Food, and Nutrition” and “Mendelian Randomization Analysis” were identified. Within the “Diet, Food, and Nutrition” hierarchy, we focused on “Food,” “Fermented Food,” and “Beverages” due to our interest in assessing the individual components of diet that affect health. The following search was completed on PubMed, “Mendelian Randomization Analysis”[Mesh] AND (“Beverages” [Mesh] OR “Fermented Foods” [Mesh] OR “Food” [Mesh]), which returned 378 articles (search date, January 24, 2025). Abstracts were reviewed by two independent researchers to identify manuscripts evaluating the relationship between a food, food group, or beverage on any health outcome. Reviews, comments, editorials, records not in English, and manuscripts evaluating nutrients were removed, leaving 178 manuscripts that were read in full. The flow diagram outlining the identification and screening process is illustrated in Supporting Information Figure S1.

### 2.2 Data Extraction and Synthesis

To summarize the common methodological practices in dietary intake MR, we extracted methodological information reported in most MR studies. This included the construction of the IV, the evaluation of the exclusion restriction assumption, and the selection of MR estimators and sensitivity tests. IV composition was classified as either consisting of variants with known biologically relevant mechanisms or variants of unknown mechanism that surpassed a statistical threshold from a GWAS. We also recorded the genetic similarity (i.e., ancestry) from which the IV was derived, as well as whether sex‐specific genetic effects were considered. The exposures were grouped into the following food groups: coffee, tea, alcohol, dairy, fruit, vegetables, and other. Studies that evaluated more than five food groups were classified generally as “diet.” Studies were classified as “other” if the food was studied in less than 5 studies such as cereal, sugar‐sweetened beverages, dark chocolate, noodles, meats, and nuts. The included studies and characteristics are provided in Supporting Information Table S1.

### 2.3 Selection of Diet and Health Outcomes

We selected four positive control relationships between foods or beverages and health outcomes that are well supported by nutrition science and have plausible biological mechanisms. This included oily fish on triglycerides (TG) (Bhatt et al. 2020) and cardiovascular disease (CVD) (Bhatt et al. 2020) and alcohol intake on alanine aminotransferase (ALT) (Alatalo et al. 2008; Ruhl and Everhart 2005) and liver cirrhosis (Lieber et al. 1965; Simpson et al. 2019), examining both a biomarker and a disease outcome for each exposure. Although there are variants in alcohol dehydrogenase enzymes known to impact alcohol consumption (Thomasson et al. 1993), there are no known variants with direct mechanistic links to oily fish intake. We used height in populations with adequate nutrition as a negative control.

### 2.4 Data Sources

Published GWAS of oily fish and overall alcohol intake in 455 K individuals of European genetic ancestry from the UK Biobank (UKB), adjusted for age and sex, and inverse normal transformed (Cole et al. 2020), were used to derive IVs, created from independent genome‐wide significant variants (r^2^ < 0.8, clumping window 500 KB).

Biomarker (ALT, TG) and disease (CVD, cirrhosis) IVs were derived from European‐ancestry GWAS that maximized sample size and avoided sample overlap with the UKB (Aragam et al. 2022; Ghouse et al. 2024; Graham et al. 2021; Pazoki et al. 2021). Height GWAS summary statistics were from Yengo et al. (2018), as the more recent publicly available GWAS on height contained sparse genome‐wide information. The IVs are presented in Supporting Information Table S2.

We harmonized dietary intake and health outcome GWAS data using custom scripts to align the effect alleles between studies, remove ambiguous variants with minor allele frequencies between 0.45 and 0.55, and identify proxies using LDLinkR and the Great Britain reference population in build 37 (r^2^ > 0.8). When proxies were needed, the dietary intake variant was also replaced for consistency (Hartwig et al. 2016). Proxies were not identified in the multivariable MR analyzes.

### 2.5 MR Estimators and Methods

The Wald ratio was used for single instrument estimates. For IVs with more than one variant, the inverse variance weighted (IVW) estimator with random effects, weighted median (WM), and MR‐Egger estimators were used (Burgess and Thompson 2017). In contrast to the IVW estimator with random effects, the WM method is more robust to the presence of directional pleiotropy and invalid variants. For the IVW tests, instrument strength was approximated using the F‐statistic, and heterogeneity was assessed with Cochran's Q statistic. We used the MR‐Egger intercept test to assess the presence of directional pleiotropy (Burgess and Thompson 2017). Steiger filtering was used to remove variants from the IV that explained more variance in the outcome than the exposure (Hemani et al. 2017). Using a conservative multiple testing adjustment, significance was set at α = 0.008 (0.05/6 trait pairs). We selected for significant associations and did not adjust the selected effects or confidence intervals.

### 2.6 PheWAS as an Approach to Filter the IV

PheWAS data were obtained from the second round of the Neale Lab UK Biobank GWAS release, including traits related to anthropometrics, biomarkers, disease status, and lifestyle (GWAS Results 2018). Binary traits with an effective sample size of 50,000 or inverse rank‐transformed quantitative traits were included (829 traits total). Traits with a genetic correlation greater than 0.75 with the dietary exposure were removed.

Using the PheWAS data, we tested two methods for filtering the IV with the goal of removing variants acting on traits correlated with dietary intake. The first method, PheWAS‐T‐test (PheWAS‐TT), removed variants from the IV if their association with any PheWAS trait was statistically stronger (in terms of variance explained) than their association with diet, based on a one‐sided t‐test comparing SNP‐trait correlations and corrected for multiple testing across traits (Darrous et al. 2024). The second method, PheWAS‐Percent Variance Explained (PheWAS‐PVE), removed variants from the IV if they explained more variance with any PheWAS trait than that of diet. Because of the thresholding of the PheWAS‐PVE method, it tended to remove more variants from the IV than the PheWAS‐TT method. PVE was calculated using GWAS summary statistics using the following formula by Shim et al (Equation 1) (Shim et al. 2015).

(1) $$\mathrm{PVE}=\frac{2\beta^{2}\mathrm{MAF}\left(1-\mathrm{MAF}\right)}{2\beta^{2}\mathrm{MAF}\left(1-\mathrm{MAF}\right)+\mathrm{se}{\left(\beta \right)}^{2}2\mathrm{NMAF}\left(1-\mathrm{MAF}\right)}.$$

### 2.7 Multivariable MR

The PheWAS‐TT method generates a list of variants and their PheWAS‐trait associations that are stronger than those with the dietary exposure of interest, suggesting they impact diet indirectly. While most MVMR analyzes utilize literature knowledge to *a priori* select traits suspected to confound the causal path, we tested a data‐driven method to select these traits. This method used summary‐level data, genetic correlation analysis, and hierarchical clustering to systematically select representative traits for inclusion in MVMR mediation analysis. For all confounding traits identified from PheWAS‐TT, pair‐wise genetic correlations were calculated using LD Score Regression (Bulik‐Sullivan et al. 2015; Results Files for LDSR of UK Biobank GWAS 2022; Schizophrenia Working Group of the Psychiatric Genomics Consortium et al. 2015). Hierarchical clustering, based on the Euclidean distance matrix, grouped these phenotypes into distinct clusters. The number of clusters was determined by visually inspecting the dendrogram for biological interpretability and by maximizing the change in branch height. For each cluster, a representative trait was selected by identifying the trait with the highest number of significant variants in common with the dietary trait. We identified GWS variants for these representative traits by clumping independent signals (r^2^ = 0.01, 10,000 kb window) using Plink (v1.9) and the 1000 Genome Project Phase 3 European‐Ancestry reference panel. MVMR models were then fit with GWS IVs, including the representative traits and the original dietary exposure simultaneously. Relying on summary‐level data, genotypic correlations were used to estimate phenotypic correlations for MVMR, as they have been shown to be highly correlated across groups of traits (Sodini et al. 2018).

### 2.8 MR‐CAUSE

We employed MR‐CAUSE, a recent method that incorporates genome‐wide data to test if the relationship between the exposure and outcome is causal and/or shared with a confounder (i.e., uncorrelated pleiotropy) (Supporting Information Figure S2) (Morrison et al. 2020). MR‐CAUSE compares the fit of the data under two models: a causal (exposure → outcome) and a shared model driven by an unknown confounder (confounder → outcome). The MR‐CAUSE nuisance parameters were calculated using 1,000,000 variants without replacement. The genome‐wide variants were pruned using the ld_prune function with the 1000 Genome CEU population and a statistical threshold of 5 × 10^−3^.

### 2.9 Software

Analyzes were performed using RStudio (v 4.2.2). The libraries MendelianRandomization (v 0.10.0) (Yavorska and Burgess 2017), MR‐CAUSE (v 1.2.0) (Morrison et al. 2020), and MVMR (v 0.4) (Burgess and Thompson 2015) were used to perform MR. Steiger filtering was performed using TwoSampleMR (v 0.6.16) (Hemani et al. 2018).

## 3 Results

### 3.1 Scoping Review of the Dietary Intake MR Literature

Our scoping review identified 178 studies that used MR to test the causal effect of foods or beverages (excluding nutrients) on health outcomes, with a notable increase each year over the last 5 years, and 76 studies (~43%) published in 2024 (Figure 1A).

**Figure 1:**
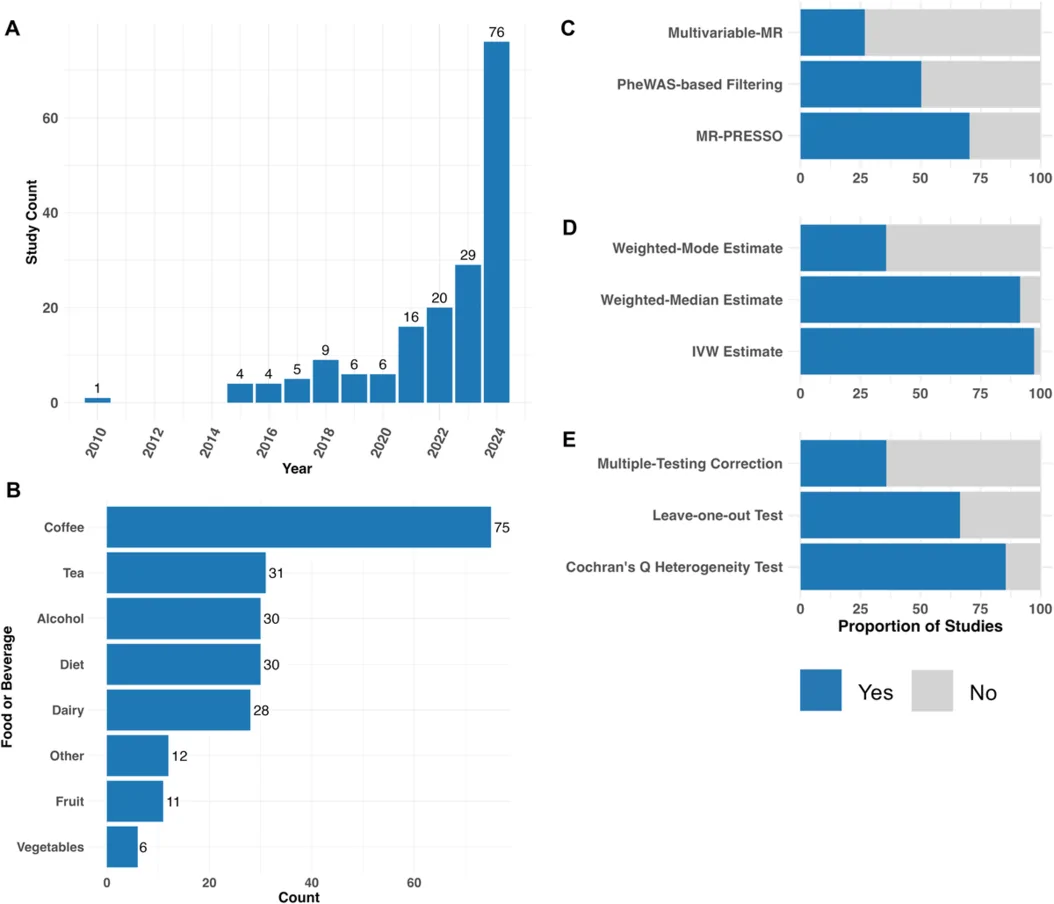
Assessment of the diet MR literature. A: Histogram showing dietary intake MR studies published by year. When the search was conducted (Jan 24, 2025) one study was published in 2025 and is not pictured. B: Distribution of the foods and beverages studied. Studies evaluating more than 5 foods are included in “Diet.” “Other” included cereal, sugar‐sweetened beverages, dark chocolate, noodles, meats, and nuts. Many studies evaluated multiple dietary exposures. C: The proportion of studies that used each method designed to address IV specificity. D: The proportion of studies that used each MR estimator. E: The proportion of studies that applied each sensitivity test.

The most common dietary exposures were coffee (*n* = 75), tea (*n* = 31), alcohol (*n* = 30), overall diet (*n* = 30), and dairy (*n* = 28) (Figure 1B). Most studies used GWS variants to build the IV (*n* = 143, 80%), most often using the standard genome‐wide significance threshold (5 × 10^−8^; *n* = 110, 77%); however, a considerable number used a more permissive threshold, such as 5 × 10^−6^. Only 20% (*n* = 35) of the studies used IVs with functional relevance to the dietary exposure, and these were most represented by dairy, coffee, tea, and alcohol. Dairy intake was commonly represented by variants in the gene *LCT*, associated with lactase persistence, while coffee and tea intake were represented by variants in genes involved in caffeine metabolism or regulation, *CYP1A1/2* and *AHR*. Alcohol intake was represented by variants affecting alcohol and aldehyde dehydrogenase genes (*ADH* and *ALDH*), respectively. However, despite the availability of functionally relevant variants influencing intake, these exposures were often represented by broader GWS IVs (Supporting Information Table S1). While biologically informed IVs reduce the likelihood of violating the exclusion restriction criteria, they remain scarce compared to the abundance of variant‐trait associations identified from GWAS.

Most manuscripts considered pleiotropy in some fashion (Figure 1C). The most common method (*n* = 105, 73%) was MR‐PRESSO, which removes variants that are statistical outliers in the causal relationship without using external data under the assumption that they are pleiotropic. However, this assumption may remove valid instruments, and therefore bias the causal estimates. In contrast, MVMR, which incorporates additional data to estimate the direct effect of diet while controlling for confounders, was only performed 28% of the time; the traits included in MVMR ranged from other dietary factors to biological traits relevant to the outcome. Socioeconomic and environmental factors like education and physical activity levels were rarely considered, although they were often adjusted for in observational (non‐genetic) analyzes when studies performed both non‐genetic and genetic analyzes. Approximately half of the studies used a PheWAS‐based method (*n* = 74, 52%), such as Phenoscanner, an online tool facilitating phenome scans that is no longer accessible (Kamat et al. 2019), or searched the GWAS catalog, to investigate pleiotropy; however, these studies inconsistently described key methodological choices, including which traits were examined for pleiotropy, how those traits were chosen, and the thresholds used to exclude variants.

Among studies that used a GWS IV (*n* = 143), the most common estimators included the inverse variance weighted (IVW; *n* = 140, 98%) and weighted median (WM; *n* = 135, 94%) estimators (Figure 1D). Most studies (*n* = 123, 86%) evaluated instrument strength using the F‐statistic, with the common threshold of F > 10 considered reasonably strong. Sensitivity analyzes, including the leave‐one‐out method (*n* = 100, 70%) and calculating Cochran's Q‐statistic (*n* = 127, 89%), were frequently used to evaluate heterogeneity (Figure 1E). Other methods to evaluate heterogeneity were cited less frequently, including the use of funnel plots (*n* = 54, 38%).

Most dietary intake MR studies have been based on individuals of European ancestry (*n* = 158, 89%), reflecting the biases in the availability of GWAS summary statistics. Additionally, no studies utilized sex‐specific IVs for diet, even when using sex‐specific genetic effects for outcomes such as ovarian or prostate cancer (*n* = 22, 12%).

### 3.2 Diet‐Health Associations

The data sources for the exposures and outcomes are listed in Table 1.

**Table 1:** Description of the exposure and outcome GWAS data sources used in the MR analyzes.

Trait		Data source	Ancestry	Sample size
Dietary intake exposure	Oily fish intake	Cole, 2020, UK Biobank	European	446,854
	Alcohol intake	Cole, 2020, UK Biobank	European	446,854
Health outcome	Triglycerides (TG)	Graham, 2021, GLGC	European	1,320,016
	Cardiovascular disease (CVD)	Aragam, 2022, 9 cohorts	European	181,522 cases and 984,168 controls
	Alanine aminotransferase (ALT)	Pazoki, 2021, UK Biobank	European	437,267
	Liver cirrhosis	Ghouse, 2024, 9 cohorts	European	15,225 cases and 1,564,786 controls
	Height	Yengo, 2018, GIANT and UK Biobank	European	709,714

Abbreviations: GIANT, Genetic Investigation of ANthropometric Traits; GLGC, Global Lipids Genetics Consortium.

Because many foods and beverages do not have biologically informed IVs, GWS IVs are more commonly used in the dietary intake MR literature. Therefore, we tested if the use of external phenome‐wide genetic associations via PheWAS could reduce pleiotropy from the GWS‐IVs, in the absence of biologically informed IVs. For alcohol, we compared these results with those from a biologically informed IV MR.

#### 3.2.1 Oily Fish

Using the GWS‐IV, a significant relationship was identified between oily fish and TG by the WM but not the IVW estimator (WM: ß = − 0.12 [95% CI −0.16 to −0.07], P = 1.87 × 10^−6^) (Figure 2A). Similarly, filtering the IV using PheWAS‐PVE, the relationship was identified by the WM (oily fish on TG, WM: ß = − 0.13 [95% CI −0.20 to −0.058], P = 3.09 × 10^−4^) but not IVW estimator. When filtering by PheWAS‐TT, the expected causal effect was identified by IVW and WM (IVW: ß = − 0.12 [95% CI −0.18 to −0.054], P = 2.36 × 10^−4^; WM: ß = − 0.14 [95% CI −0.18 to −0.086], P = 4.32 × 10^−8^). No significant relationships were identified between oily fish and CVD (Figure 2B). A significant inverse association was identified between oily fish and height following PheWAS‐PVE filtering (WM: ß = − 0.25 [95% CI −0.33 to −0.17], P = 2.21 × 10^−10^) (Figure 2C).

**Figure 2:**
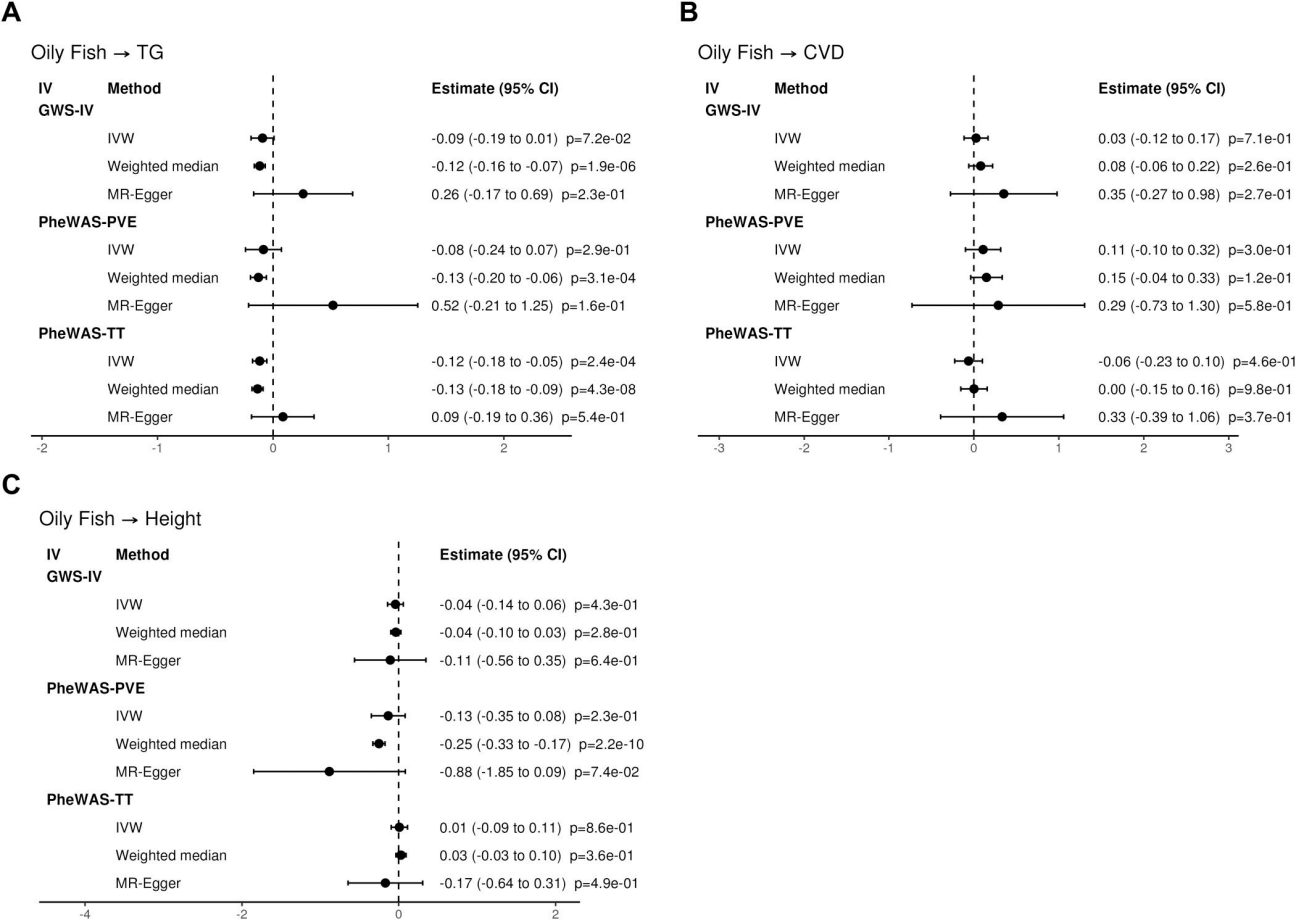
Genetic associations by different IVs for oily fish on A: triglyceride concentration, B: cardiovascular disease, and C: height. The whiskers represent 95% confidence intervals. IVW, inverse variance weighted; WM, weighted median.

The PheWAS‐PVE method, based on strict thresholding, removed more variants than the PheWAS‐TT method, based on statistical differences, but was less effective at reducing heterogeneity (Supporting Information Table S3). For the association between oily fish and TG, the GWS‐IV had 75 variants and a Q‐statistic of 1370, which were reduced to 59 variants and a Q‐statistic of 322 by the PheWAS‐TT method, and 47 variants and a Q‐statistic of 1270 by the PheWAS‐PVE method. The substantial reduction in heterogeneity by PheWAS‐TT indicated greater effectiveness in identifying variants to exclude from the IV and led us to focus our downstream analyzes on the output of the PheWAS‐TT method. The removed variants and their associated traits were dominated by anthropometric measurements such as fat mass, heel bone mineral density, and blood count (Supporting Information Table S4). For all IVs, the F‐statistic ranged from 40 to 45 indicating adequate instrument strength.

#### 3.2.2 Alcohol

Using the GWS‐IV, the relationship between alcohol and ALT was statistically significant, but in the opposite direction of what was expected (IVW: ß = − 0.02 [95% CI −0.035 to −0.008], P = 1.7 × 10^−3^; WM: ß = − 0.02 [95% CI −0.03 to −0.01], P = 4.55 × 10^−4^) (Figure 3A). No significant relationships were identified between alcohol and liver cirrhosis after multiple testing correction (Figure 3B). A nominal effect of alcohol and height (WM: ß = 0.073, [95% CI 0.003 to 0.14], *p* = 0.04) was identified and persisted following PheWAS‐PVE but not PheWAS‐TT filtering (Figure 3C).

**Figure 3:**
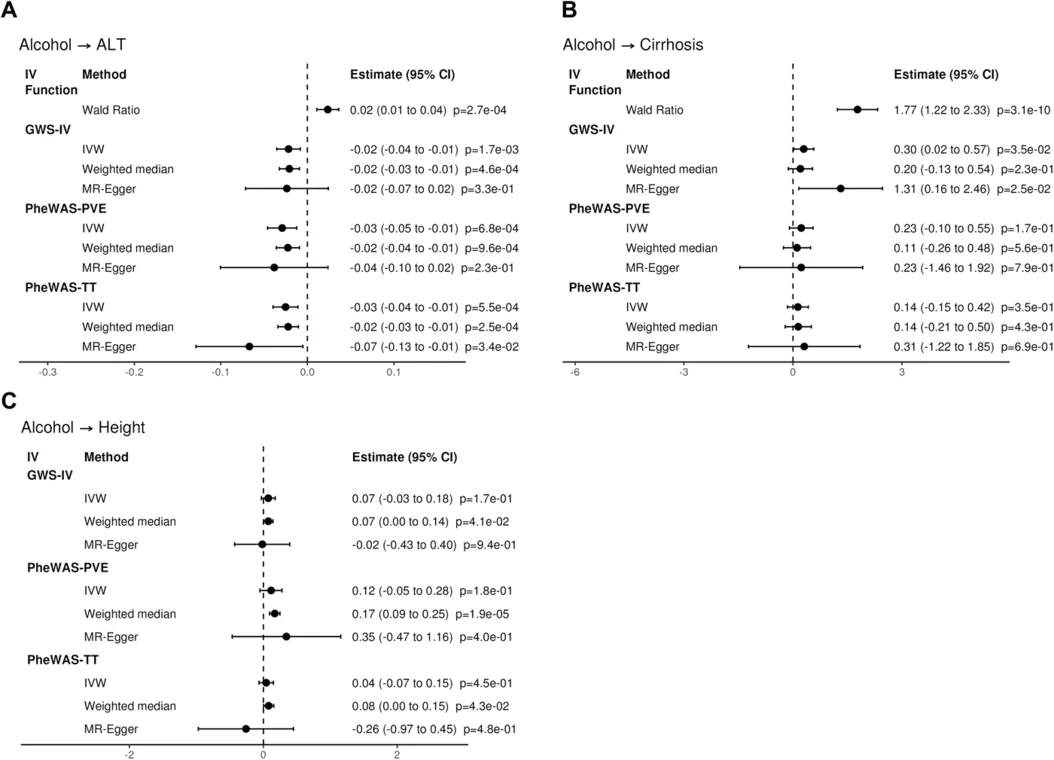
Genetic associations by different IVs for alcohol on A: alanine aminotransferase concentration, B: liver cirrhosis, and C: height. The whiskers represent 95% confidence intervals. IVW, inverse variance weighted; WM, weighted median.

The *ADH1B* rs1229984 (C > T) variant increases the rate of ethanol metabolism resulting in the build‐up of acetaldehyde and unpleasant biological effects such as facial flushing (Thomasson et al. 1993). Using this functional instrument, we identified significant relationships in the expected direction between alcohol and ALT (Wald ratio: ß = 0.02, [95% CI 0.01–0.04], P = 2.7 × 10^−4^) and liver cirrhosis (ß = 1.77, [95% CI 1.22–2.33], P = 3.09 × 10^−10^) (Figure 3A,B). Neither the functional variant nor a proxy in high LD was present in the height GWAS.

As with oily fish, the PheWAS‐TT removed fewer variants from the GWS‐IV than the PVE method while reducing heterogeneity (Supporting Information Table S3) reinforcing that the PheWAS‐TT method filtered variants more effectively than the PVE method. The F‐statistic ranged from 35 to 45 indicating adequate instrument strength for all IVs. We also tested the functionally informed variant rs1229984 by the PheWAS methods and observed that it was retained by both approaches.

### 3.3 PheWAS Informed Trait Selection for MVMR

In addition to filtering the IV to remove confounding, we tested MVMR to instead control for confounders in the MR model. Using a data‐driven approach, we objectively identified traits using the PheWAS‐TT output. For oily fish and alcohol exposures, highly polygenic anthropometric and blood count traits were selected for inclusion in the MVMR. This resulted in large IVs that were weakly associated with dietary intake (F‐statistic range: 4.5−5.9) and highly heterogeneous (Q range: 2286−24,737). MVMR did not identify any of the expected relationships (significance and direction of effect) between dietary intake and the health outcomes (Supporting Information Table S5).

### 3.4 MR‐CAUSE

Due to the polygenic genetic architecture influencing dietary intake, the lack of variants with large effect sizes, and suspected widespread pleiotropy, we employed MR‐CAUSE, a genome‐wide approach designed to handle these factors. MR‐CAUSE model comparisons indicated that both the sharing and the causal models fit the data significantly better than the null models for oily fish and TG, and alcohol and ALT (Table 2). For both trait pairs, the causal model provided a significantly better fit than the shared model (ΔELPD sharing vs causal < 0, *p* < 0.008), supporting a causal effect of diet in addition to correlated pleiotropy (ΔELPD null vs. sharing < 0, *p* < 0.008). The causal model estimated oily fish to decrease TG (γ = −0.08 [−0.11, −0.05]) and alcohol to decrease ALT (γ = −0.01 [−0.02, −0.01]) (Table 3). Evidence of confounding contrasted with the findings of the commonly used MR‐Egger intercept method, which found no evidence of directional pleiotropy (*p* > 0.075 in all cases, Supporting Information Table S6), suggesting that while there is no directional pleiotropy, on average, the relationship between the IV and outcome can be explained by correlated pleiotropy due to a shared heritable factor.

**Table 2:** MR‐CAUSE results demonstrating the fit of the shared and causal models for each positive and negative control. The statistics represent the comparison of model 1 and model 2. Negative ΔELPD values indicate model 2 fits the data better than model 1.

Dietary trait	Outcome	Model 1	Model 2	ΔELPD	Standard error of ΔELPD	Z	*p*‐value
Oily fish	Triglycerides	Null	Sharing	−14.554	3.436	−4.235	1.14E‐05
		Null	Causal	−21.366	5.074	−4.211	1.27E‐05
		Sharing	Causal	−6.812	1.649	−4.131	1.81E‐05
	Cardiovascular disease	Null	Sharing	0.476	0.041	11.613	1
		Null	Causal	1.263	0.083	15.198	1
		Sharing	Causal	0.787	0.066	11.991	1
	Height	Null	Sharing	−0.060	0.461	−0.130	0.448
		Null	Causal	−1.697	2.108	−0.805	0.210
		Sharing	Causal	−1.636	1.656	−0.988	0.161
Alcohol	Alanine aminotransferase	Null	Sharing	−8.006	2.964	−2.701	0.003
		Null	Causal	−12.544	4.708	−2.664	0.004
		Sharing	Causal	−4.538	1.774	−2.558	0.005
	Liver cirrhosis	Null	Sharing	0.424	0.302	1.403	0.920
		Null	Causal	1.061	0.730	1.453	0.927
		Sharing	Causal	0.637	0.440	1.447	0.926
	Height	Null	Sharing	−2.312	1.524	−1.517	0.065
		Null	Causal	−5.475	3.327	−1.646	0.050
		Sharing	Causal	−3.163	1.834	−1.724	0.042

**Table 3:** MR‐CAUSE results demonstrating the estimated causal effect (γ), shared effect (η), and uncorrelated effect (q) in the shared and causal models for each positive and negative control. In the shared model, the causal effect is set to 0. * represents model significance as indicated in Table 2.

Dietary trait	Outcome	Model	Gamma, γ	Eta, η	q
Oily fish	Triglycerides	Sharing*	NA	−0.11 (−0.17, −0.07)	0.49 (0.24, 0.71)
		Causal*	−0.08 (−0.11, −0.05)	0 (−0.31, 0.3)	0.03 (0, 0.25)
	Cardiovascular disease	Sharing	NA	−0.01 (−0.79, 0.79)	0.03 (0, 0.22)
		Causal	0 (−0.07, 0.06)	0 (−0.72, 0.72)	0.04 (0, 0.25)
	Height	Sharing	NA	0.14 (−0.33, 0.79)	0.05 (0, 0.31)
		Causal	0.05 (0, 0.09)	0.01 (−0.57, 0.69)	0.03 (0, 0.25)
Alcohol	Alanine aminotransferase	Sharing*	NA	−0.02 (−0.04, −0.01)	0.31 (0.09, 0.55)
		Causal*	−0.01 (−0.02, −0.01)	0 (−0.14, 0.09)	0.03 (0, 0.24)
	Liver cirrhosis	Sharing	NA	0.32 (−1.2, 2.48)	0.03 (0, 0.22)
		Causal	0.03 (−0.1, 0.15)	0.11 (−1.29, 1.86)	0.04 (0, 0.25)
	Height	Sharing	NA	0.19 (0.01, 0.56)	0.16 (0.01, 0.43)
		Causal	0.07 (0.03, 0.11)	0.01 (−0.59, 0.61)	0.03 (0, 0.25)

## 4 Discussion

The availability of GWAS summary statistics and the software to perform analyzes has contributed to the rapid rise of MR studies and fueled concern about the credibility of causal estimates from dietary intake MR (Burgess et al. 2024). To disentangle this rapidly evolving field, we performed a scoping review of the methods commonly used in this field, which revealed that many dietary intake MR use IVs based on genome‐wide significant associations instead of IVs with known biological relevance. IVs composed of variants with unknown biological function are more difficult to evaluate for potential violations of the exclusion restriction assumption because the mechanisms through which they influence the outcome are less well understood. Thus, we sought to test if PheWAS approaches could be used to filter the GWS IV to better capture the direct effects of dietary intake on health outcomes supported by nutrition science.

When conducting MR analyzes of dietary intake, genetic variants that influence intake behavior—such as variants affecting lactase persistence, sensory perception, or caffeine and ethanol metabolism—are particularly informative (Enattah et al. 2002; Hwang et al. 2026; Sachse et al. 1999; Thomasson et al. 1993). Although these functional variants often explain less variance than genome‐wide significant instruments derived from large GWAS, they are more likely to satisfy core MR assumptions by having a clearer biological link to the exposure and by being more able to rule out horizontal pleiotropy. Nevertheless, functional variants are still vulnerable to paradoxical situations, which require careful interpretation to avoid misconstruing the MR results such as variants that disrupt the normal function of the exposure or are associated with multiple biomarkers on separate biological pathways (Holmes et al. 2017). Additionally, because relatively few functional variants are known to directly influence dietary behaviors, MR may be better suited to evaluate the health effects of circulating nutrients rather than whole‐food exposures.

In this context, asking whether circulating omega‐3 fatty acid levels influence triglyceride concentrations may represent a more appropriate MR question than asking whether oily fish intake affects triglycerides, because functional variants in fatty acid desaturase genes (*FADS1/2*) have been identified that alter omega‐3 levels (Loukil et al. 2024). Findings from such analyzes can provide mechanistic evidence that complements dietary research using other study designs and may inform public health strategies aimed at modifying nutrient status.

Although functional variants satisfy the relevance assumption, they may still violate the exclusion restriction assumption through horizontal pleiotropy arising from known or unknown biological mechanisms. The variant rs1229984 in *ADH1B* is best known for altering alcohol metabolism, yet it is associated with numerous traits in the GWAS Catalog. Many of these associations are plausibly downstream consequences of altered alcohol consumption (i.e., vertical pleiotropy) and therefore would not violate the exclusion restriction assumption. However, mediation analyzes have identified associations with esophageal and colorectal cancer that appear to be at least partially independent of alcohol consumption (Crous‐Bou et al. 2013; Lewis and Smith 2005), demonstrating that even well‐characterized functional variants can exert effects through alternative pathways. Although these cancer‐related effects are unlikely to influence the relationship between alcohol intake and liver health, they underscore the importance of systematically evaluating potential pleiotropic pathways and interpreting MR results alongside results from other study designs.

We tested PheWAS filtering approaches for situations where functional IVs are missing to identify and exclude phenotypes that could plausibly introduce horizontal pleiotropy, while recognizing that no strategy can completely eliminate the possibility of unknown pleiotropic effects. Although filtering via the PheWAS‐TT reduced heterogeneity and increased instrument strength versus the PVE method, both failed to identify the direct relationship between alcohol and ALT identified by the functional rs1229984 instrument. Future efforts to refine the IV should focus on identifying biologically meaningful variants instead of applying data‐driven filtering approaches to the GWS‐IVs.

Others have raised the concern that genetic loci associated with dietary intake are partly driven by indirect effects from confounding traits and developed nuanced workflows requiring individual‐level data to address this (Pirastu et al. 2022). For example, MR IVs have been identified using a multi‐step approach, in which GWAS are first performed using a Bayesian estimation to account for the effect of other traits on dietary intake. Then, variants are included in the IV if there is statistical evidence to support their direct effect on diet based on the magnitude of change in the effect when covariates are included in the analyzes (Taba et al. 2021). This effort revealed that the effect sizes of loci associated with diet are altered when adjusting for the confounders, such as BMI and education level, implying that these factors should be considered when estimating the effects of diet on health with MR.

*Limitations*. Due to data availability, linkage disequilibrium, and minor allele frequency, these analyzes were restricted to GWAS conducted in individuals with European genetic ancestry. We originally sought to compare the effects of the lifelong genetically predicted exposures in MR to RCT studies in shorter windows. We did not achieve this due to the ordinal nature of the diet traits, coupled with the difficulty of identifying RCTs solely testing the food or beverage of interest. Our selection of positive controls was constrained by the limited availability of well‐powered GWAS summary statistics for dietary exposures, as large‐scale studies are currently available for only a subset of foods such as those assessed by the 29‐item food frequency questionnaire in the UK Biobank (Bradbury et al. 2018). We analyzed the causal effects of oily fish on TG and CVD because omega‐3 fatty acids, a major nutrient in oily fish, consistently lower TG and have long been hypothesized to reduce CVD, although more recent evidence has challenged the magnitude and consistency of their CVD benefits (Nicholls et al. 2020). We expected that social determinants of health (SDOH) would confound the IVs, yet the PheWAS‐TT method mostly identified anthropometric traits or biomarkers. This may be due to the unequal representation of SDOH traits in our PheWAS data, the polygenicity of the traits, or the precision with which anthropometric traits are measured versus SDOH traits and behavioral factors like diet obtained from dietary recall questionnaires. It is also possible that the relevant traits were not included in the PheWAS traits analyzed. Nevertheless, it is important to point out that traits that are genetically correlated, such as diet and SDOH, may not be strongly associated with the top diet loci.

## 5 Conclusion

Concern about the credibility of dietary intake‐MR is timely, given the growing interest and proliferation of publications in this field. In the absence of biologically informed IVs, PheWAS based approaches cannot be relied on to isolate the direct effect of dietary intake on health.

## Conflicts of Interest

The authors declare no conflicts of interest.

## Supporting information


**Figure S1**: Diagram of the studies identified and included from the assessment of the dietary intake MR literature up to January 24, 2025. Mesh, Medical Subject Headings; IV, instrumental variable

**Figure S2:** Example models demonstrating the causal and shared models from MR‐CAUSE. C, confounder; IV, instrumental variable; ALT, alanine aminotransferase

Supporting File S3

Supporting File S4

## Data Availability

All data used in these analyzes is publicly available. Code to recreate these analyzes is available at https://github.com/bytesizesci/refine_GI_diet_MR.
