# Supplementary material for: Refining Genetic Instruments for Dietary Intake Mendelian Randomization Using Phenome‐Wide Association Studies
Source: Genet Epidemiol. 2026 Oct 1;50(8):e70059. doi: 10.1002/gepi.70059 (PMC13628697; doi:10.1002/gepi.70059)
Supplement: Supplementary file 1 [file GEPI-50-0-s001.pdf]

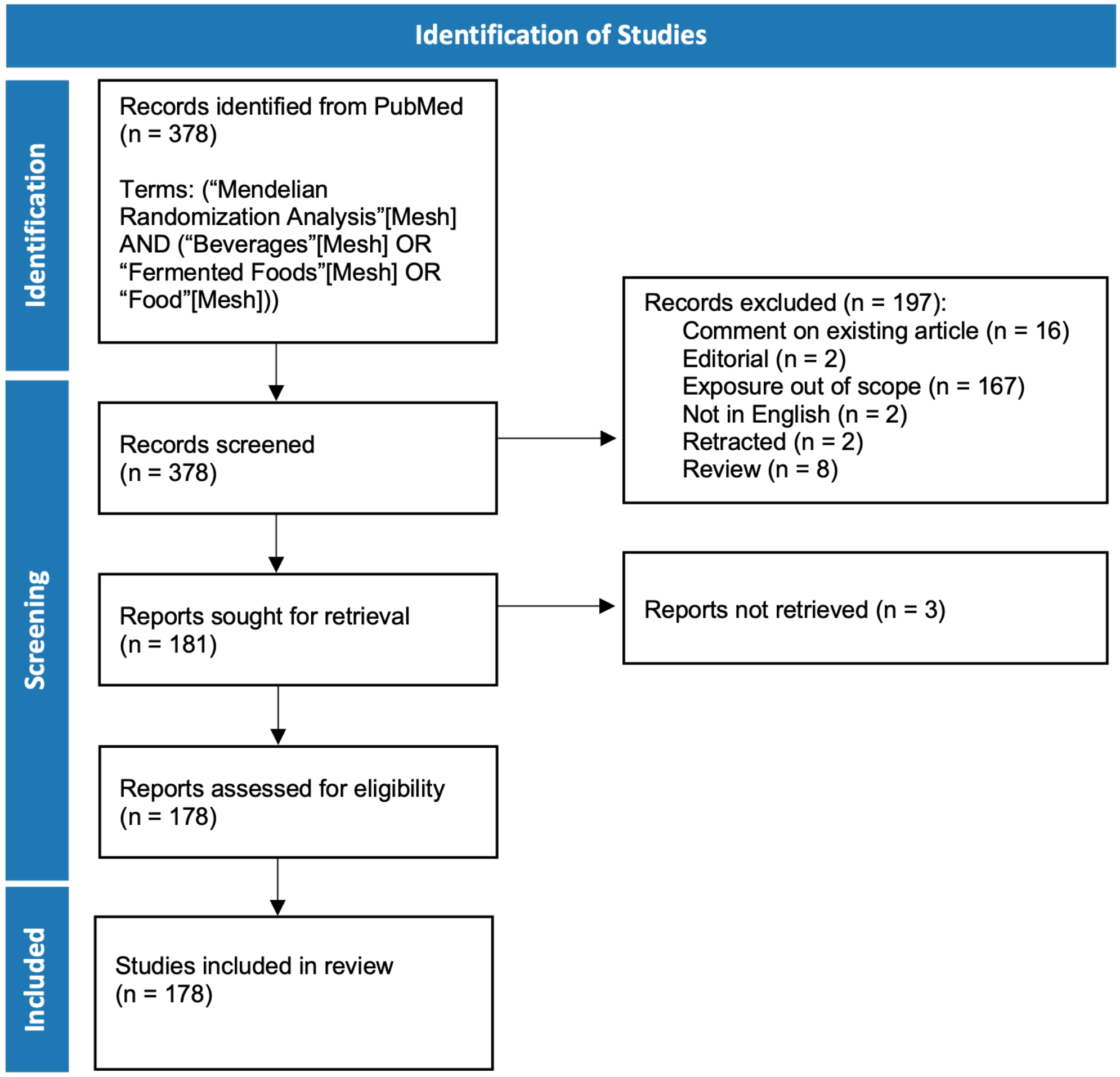

Supplemental Figure 1. Diagram of the studies identified and included from the assessment of the dietary intake MR literature up to January 24, 2025. Mesh, Medical Subject Headings; IV, instrumental variable.
