# Supplementary material for: Refining Genetic Instruments for Dietary Intake Mendelian Randomization Using Phenome‐Wide Association Studies
Source: Genet Epidemiol. 2026 Oct 1;50(8):e70059. doi: 10.1002/gepi.70059 (PMC13628697; doi:10.1002/gepi.70059)
Supplement: Supplementary file 2 [file GEPI-50-0-s003.pdf]

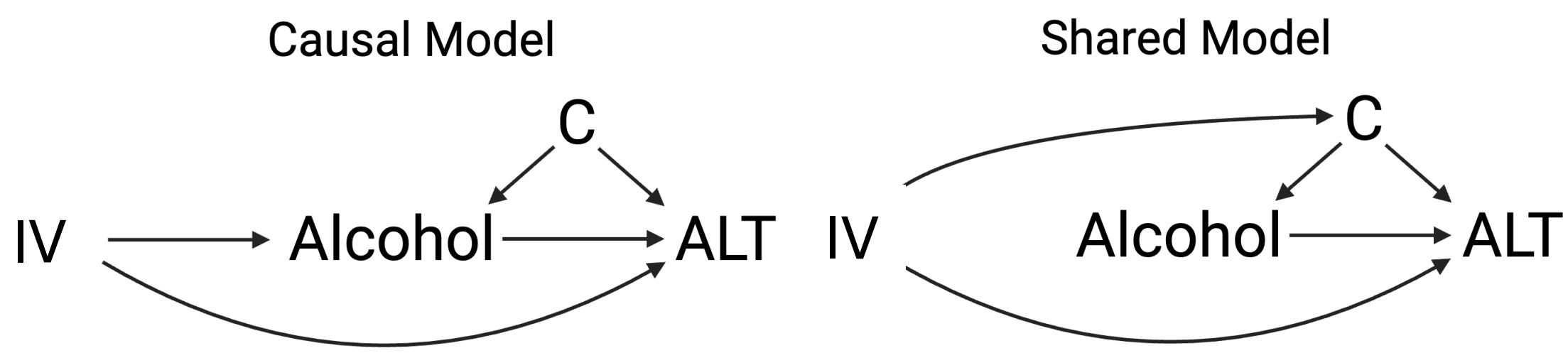

Supplemental Figure 2. Example models demonstrating the causal and shared models from MR-CAUSE. C, confounder; IV, instrumental variable; ALT, alanine aminotransferase.
