# Supplementary material for: Refining Genetic Instruments for Dietary Intake Mendelian Randomization Using Phenome‐Wide Association Studies
Source: Genet Epidemiol. 2026 Oct 1;50(8):e70059. doi: 10.1002/gepi.70059 (PMC13628697; doi:10.1002/gepi.70059)
Supplement: Supplementary file 3 [file GEPI-50-0-s004.pdf]

STROBE-MR checklist of recommended items to address in reports of Mendelian randomization studies[1 2]

Item No.	Section	Checklist item	Page No.	Relevant text from manuscript
1	**TITLE and ABSTRACT**	Indicate Mendelian randomization (MR) as the study's design in the title and/or the abstract if that is a main purpose of the study	2	Title: Refining Genetic Instruments for Dietary Intake Mendelian Randomization Using Phenome-Wide Association Studies<br><br>Abstract: Most Mendelian randomization (MR) of dietary intake use the full set of genome-wide significant (GWS) variants in the instrumental variable (IV), likely biasing causal estimates due to pleiotropy. To characterize the common methods to handle pleiotropy in dietary intake MR, we conducted a scoping review of the literature on dietary intake MR studies. We extracted information on IV construction, assessment of pleiotropy, and sensitivity analyses revealing that only 20% of studies used an IV with functional plausibility. In the absence of functionally-informed IVs, we tested if two-sample MR using GWS variants filtered for pleiotropic associations through phenome-wide association studies (PheWAS) could identify diet–health relationships supported by existing nutrition science, focusing on oily fish and alcohol intake, the latter of which has a functionally-informed IV for comparison (rs1229984 in the ADH1B gene). To further explore this question, we performed multivariable MR and employed MR-CAUSE. The numerous models consistently supported that oily fish reduced triglycerides. In contrast, GWS and PheWAS-filtered IVs suggested that alcohol decreased alanine aminotransferase levels, whereas the functional IV (rs1229984) found the opposite expected relationship. Isolating the direct effect of dietary intake from GWS IV remains challenging. Future work should focus on identifying functional variants impacting dietary behavior.
	**INTRODUCTION**			
2	**Background**	Explain the scientific background and rationale for the reported study. What is the exposure? Is a potential causal relationship between exposure and outcome plausible? Justify why MR is a helpful method to address the study question	3	Background and rationale: Genetic instruments for dietary intake traits are publicly available and MR of these traits on health outcomes is increasingly common. However, dietary intake is affected by both genetics and environmental factors, and therefore, it is likely that many of the variants in the instrumental variable (IV) affect diet through indirect paths potentially biasing the causal estimates.<br><br>Exposure: The exposures included oily fish intake (ordinal, described by frequency from never to daily intake) and alcohol intake (continuous, described by never to daily intake). The exposures are described in detail in Table 1.<br><br>Plausibility: Diet has profound effects on health, but many diet-health relationships are unsettled. With the availability of IVs for dietary intake traits (food groups, foods, food liking, etc.) dietary intake MR are becoming more common. However, dietary intake is influenced by non-genetic factors such as socioeconomic and health status. It is possible that many of the

				variants statistically associated with diet act through indirect paths, potentially violating the exclusion-restriction assumption.
3	**Objectives**	State specific objectives clearly, including pre-specified causal hypotheses (if any). State that MR is a method that, under specific assumptions, intends to estimate causal effects	3	Lines 80-87: Given the limitations of functional IVs, the recent availability of numerous and large dietary intake GWAS, and the ease of performing two-sample MR analyses with user-friendly R packages and online portals, a surge of MR studies have occurred – often at the expense of the exclusion restriction assumption. Therefore, we first conducted a brief critical assessment of the dietary intake MR literature to identify common methods for building the IV and estimating the causal relationships. Second, we tested if, in the absence of known functionally informed IVs, we could reproduce diet-health relationships supported by nutrition science with data-driven approaches that use external GWAS data and PheWAS to filter the IV.<br>Lines 62-65: In order for MR to accurately estimate causal effects, three main conditions must be satisfied: 1) relevance: the IV is associated with the exposure; 2) exchangeability: the IV is independent of confounders of the exposure-outcome relationship; and 3) exclusion restriction: there are no paths from the IV to the outcome that do not go through the exposure.
	**METHODS**			
4	**Study design and data sources**	Present key elements of the study design early in the article. Consider including a table listing sources of data for all phases of the study. For each data source contributing to the analysis, describe the following:		
	a)	Setting: Describe the study design and the underlying population, if possible. Describe the setting, locations, and relevant dates, including periods of recruitment, exposure, follow-up, and data collection, when available.	4	Rationale for selecting the exposures and outcomes is given in the methods section, “Selection of diet and health outcomes.”<br>Lines 346-353: We selected four positive control relationships between foods or beverages and health outcomes that are well supported by nutrition science and have plausible biological mechanisms. This included oily fish on triglycerides (TG) and cardiovascular disease (CVD) and alcohol intake on alanine aminotransferase (ALT) and liver cirrhosis, examining both a biomarker and a disease outcome for each exposure. Although there are variants in alcohol dehydrogenase enzymes known to impact alcohol consumption, there are no known variants with direct mechanistic links to oily fish intake. We used height in populations with adequate nutrition as a negative control.<br>The genome-wide association study (GWAS) summary statistic data for exposures and outcomes are described in the methods section, “Data Sources.”<br>Lines 354-357: Published GWAS of oily fish and overall alcohol intake in 455K individuals of European genetic ancestry from the UK Biobank (UKB), adjusted for age and sex, and inverse

			normal transformed, were used to derive IVs, created from independent genome-wide significant variants ($r^2 < 0.8$, clumping window 500 KB).
b)	Participants: Give the eligibility criteria, and the sources and methods of selection of participants. Report the sample size, and whether any power or sample size calculations were carried out prior to the main analysis	4-5	Summary statistic data was used from publicly available sources. Thus, decisions about eligibility criteria were not completed for this study. We checked the covariates, transformations, and eligibility requirements from the published GWAS to ensure we selected data with minimum risk of collider bias.
c)	Describe measurement, quality control and selection of genetic variants	4-5	Quality control and harmonization is described on lines 362-366: We harmonized dietary intake and health outcome GWAS data using custom scripts to align the effect alleles between studies, remove ambiguous variants with minor allele frequencies between 0.45 and 0.55, and identify proxies using LDLinkR and the Great Britain reference population in build 37 ($r^2 > 0.8$). When proxies were needed, the dietary intake variant was also replaced for consistency. Proxies were not identified in the multivariable MR analyses.<br><br>We tested different PheWAS methods to filter the genome-wide significant IV. The PheWAS filtering methods are described in lines 378-393: PheWAS data were obtained from the second round of the Neale Lab UK Biobank GWAS release, including traits related to anthropometrics, biomarkers, disease status, and lifestyle. Binary traits with an effective sample size of 50,000 or inverse rank-transformed quantitative traits were included (829 traits total). Traits with a genetic correlation greater than 0.75 with the dietary exposure were removed.<br><br>Using the PheWAS data, we tested two methods for filtering the IV with the goal of removing variants acting on traits correlated with dietary intake. The first method, PheWAS-T-test (PheWAS-TT), removed variants from the IV if their association with any PheWAS trait was statistically stronger (in terms of variance explained) than their association with diet, based on a one-sided t-test comparing SNP-trait correlations and corrected for multiple testing across traits. The second method, PheWAS-Percent Variance Explained (PheWAS-PVE), removed variants from the IV if they explained more variance with any PheWAS trait than that of diet. Because of the thresholding of the PheWAS-PVE method, it tended to remove more variants from the IV than the PheWAS-TT method. PVE was calculated using GWAS summary statistics using the following formula by Shim et al (equation 1).
d)	For each exposure, outcome, and other relevant variables, describe methods of assessment and diagnostic criteria for diseases		The exposure and outcome data are provided in Table 1.<br><br>The exposures included oily fish intake (ordinal, described by frequency from never to daily intake) and alcohol intake (continuous, described by never to daily intake).<br><br>The outcomes included triglycerides (mg/dL), cardiovascular disease (cohort dependent, see Supplementary Table 1 of PMID 36474045), alanine aminotransferase (U/L), liver cirrhosis (ICD-9 and ICD-10 codes, logistic regression), and height (cm).

		e)	Provide details of ethics committee approval and participant informed consent, if relevant		NA
5	**Assumptions**		Explicitly state the three core IV assumptions for the main analysis (relevance, independence and exclusion restriction) as well assumptions for any additional or sensitivity analysis	3	The three core IV assumptions are stated in the introduction on lines 62-65:<br>In order for MR to accurately estimate causal effects, three main conditions must be satisfied: 1) relevance: the IV is associated with the exposure; 2) exchangeability: the IV is independent of confounders of the exposure-outcome relationship; and 3) exclusion restriction: there are no paths from the IV to the outcome that do not go through the exposure.
6	**Statistical methods: main analysis**		Describe statistical methods and statistics used		
		a)	Describe how quantitative variables were handled in the analyses (i.e., scale, units, model)	7	The exposure and outcome data are provided in Table 1.<br>The exposures included oily fish intake (ordinal, described by frequency from never to daily intake) and alcohol intake (continuous, described by never to daily intake). Exposures were adjusted by age and sex and inverse rank normalized transformed.<br>The outcomes included triglycerides (mg/dL, inverse rank normalized transformed, adjusted by age), cardiovascular disease (binary, adjusted by birthyear, sex), alanine aminotransferase (U/L, log10 transformed, adjusted by age and sex), liver cirrhosis (binary, adjusted by age and sex), and height (cm, inverse rank normalized transformed, adjusted by age and sex).
		b)	Describe how genetic variants were handled in the analyses and, if applicable, how their weights were selected	4-5	We tested several IVs for each exposure-outcome pair. The genome-wide significant variant consisted of variants statistically associated with the exposure ($P<5x10^{-8}$). We then tested two PheWAS filtering approaches and a functionally informed variant for alcohol.
		c)	Describe the MR estimator (e.g. two-stage least squares, Wald ratio) and related statistics. Detail the included covariates and, in case of two-sample MR, whether the same covariate set was used for adjustment in the two samples	5	We employed the inverse variance weighted, weighted median, and MR-Egger methods. For instruments consisting of one variant, we used the Wald ratio. We also employed MR-CAUSE. This is described in the methods section, “MR estimators and methods,” lines 367-377.
		d)	Explain how missing data were addressed	4-5	When a variant from the IV was missing in the outcome data, we attempted to find a proxy using the following strategy.<br>Proxy variants in linkage disequilibrium (r^2>0.8) with the original exposure variant were identified for variants that were missing in the outcome GWAS. In these cases, the exposure

				variant was also replaced with the proxy to avoid problems of phasing. The R package LDLinkR was used with the Great Britain reference population in build 37. When multiple proxies with identical r^2 values were available, the variant closest to the original was selected. If the variant was not in the 1000G reference panel, it was removed from the IV.
		e) If applicable, indicate how multiple testing was addressed	5	We evaluated 6 relationships. Thus, we employed a multiple testing correction of P<0.05/6=0.008. This is described on lines 375-377.
7	**Assessment of assumptions**	Describe any methods or prior knowledge used to assess the assumptions or justify their validity	4-7	Addressing the exclusion restriction assumption, we tested various IV filtering methods using external PheWAS data and multivariable MR.
8	**Sensitivity analyses and additional analyses**	Describe any sensitivity analyses or additional analyses performed (e.g. comparison of effect estimates from different approaches, independent replication, bias analytic techniques, validation of instruments, simulations)	4-7	An overarching aim of our study was to test if we could identify diet-health relationships supported by nutrition science in the absence of functionally informed IVs. Thus, we purposefully selected relationships with nutrition science support to serve as “positive controls.” For each pair, we tested various IVs and compared the effect direction and significance to the literature-based expectation.<br>We included several estimators, report heterogeneity, instrument strength in the manuscript and plotted the effects and conducted leave-one-out analyses (not reported).<br>We performed a PheWAS sensitivity analysis upon which we removed all diet-related traits from the PheWAS and repeated the analyses. The results remained stable, so we proceeded to report only the full PheWAS analysis.
9	**Software and pre-registration**			
		a) Name statistical software and package(s), including version and settings used	6	The statistical software and packages used are described in the methods including specific settings for the functions within the R packages MendelianRandomization and MRCAUSE.<br>RStudio (v 4.2.2)<br>R packages: MendelianRandomization (v 0.10.0), MR-CAUSE (v 1.2.0)<br>IVW: random effects and default setting<br>MR-CAUSE: the nuisance parameters were calculated using 1,000,000 variants without replacement. The genome-wide variants were pruned with the ld_prune function with the 1000 Genome CEU population and a statistical threshold of 5E-3.<br>Code is available at https://github.com/bytesizesci/refine_GI_diet_MR.
		b) State whether the study protocol and details were pre-registered (as well as when and where)		NA

	**RESULTS**				
10	**Descriptive data**				
		a)	Report the numbers of individuals at each stage of included studies and reasons for exclusion. Consider use of a flow diagram		NA
		b)	Report summary statistics for phenotypic exposure(s), outcome(s), and other relevant variables (e.g. means, SDs, proportions)	7	Data sources are listed in Table 1. The IVs are reported in Supplemental Table 2.
		c)	If the data sources include meta-analyses of previous studies, provide the assessments of heterogeneity across these studies		NA
		d)	For two-sample MR:<br>i. Provide justification of the similarity of the genetic variant-exposure associations between the exposure and outcome samples<br>ii. Provide information on the number of individuals who overlap between the exposure and outcome studies	7	The exposure and outcome data including the cohorts they were derived from are described in Table 1.<br>The exposure data is from the UK Biobank. Of the outcomes analyzed, only one (alanine aminotransferase) is composed solely of the UK Biobank. This study validated their GWAS using data obtained from the Rotterdam, Lifelines, and Million Veterans Program. This ALT GWAS was selected despite the overlap due to its size, availability, and recent publication date.<br>Overlap between the diet and triglyceride GWAS is estimated at ~17%.<br>It is difficult to estimate the overlap between the diet and cardiovascular disease, liver cirrhosis, and height GWAS as each were meta-analyses consisting of numerous cohorts including the UKB.
11	**Main results**				
		a)	Report the associations between genetic variant and exposure, and between genetic variant and outcome, preferably on an interpretable scale	7-9	The associations between the variants and the traits are reported in Supplemental Table 2.
		b)	Report MR estimates of the relationship between exposure and	7-9	The results are presented in the Diet-Health Associations section of the results and in Figures 2 and 3. The MR-CAUSE results are reported in Tables 2 and 3.

			outcome, and the measures of uncertainty from the MR analysis, on an interpretable scale, such as odds ratio or relative risk per SD difference		
		c)	If relevant, consider translating estimates of relative risk into absolute risk for a meaningful time period		NA
		d)	Consider plots to visualize results (e.g. forest plot, scatterplot of associations between genetic variants and outcome versus between genetic variants and exposure)	7-8	Forest plots are provided as Figures 2 and 3 in the results section.
12	**Assessment of assumptions**				
		a)	Report the assessment of the validity of the assumptions	8-9	Our study focused on the exclusion restriction assumption. While the MR-Egger intercept test did not find evidence of directional pleiotropy, MR-CAUSE found evidence of correlated pleiotropy.
		b)	Report any additional statistics (e.g., assessments of heterogeneity across genetic variants, such as I^2, Q statistic or E-value)	8	Heterogeneity via Cochran's Q statistic is reported from the IVW tests in Supplemental Table 3.
13	**Sensitivity analyses and additional analyses**				
		a)	Report any sensitivity analyses to assess the robustness of the main results to violations of the assumptions		NA
		b)	Report results from other sensitivity analyses or additional analyses	8	In addition to filtering the IV to remove confounding, we tested MVMR to instead control for confounders in the MR model. Using a data-driven approach, we objectively identified traits using the PheWAS-TT output. For oily fish and alcohol exposures, highly polygenic

					anthropometric and blood count traits were selected for inclusion in the MVMR. This resulted in large IVs that were weakly associated with dietary intake (F-statistic range: 4.5 - 5.9) and highly heterogeneous (Q range: 2,286 - 24,737). MVMR did not identify any of the expected relationships (significance and direction of effect) between dietary intake and the health outcomes (Table S5).
		c)	Report any assessment of direction of causal relationship (e.g., bidirectional MR)	5	We included Steiger filtering to remove variants from the IV if there was evidence that they acted on the outcome to the exposure.
		d)	When relevant, report and compare with estimates from non-MR analyses		We compared the direction and significance of the MR results to the nutrition-science supported relationships.
		e)	Consider additional plots to visualize results (e.g., leave-one-out analyses)		Leave-one-out plots were generated but are not presented in the manuscript.
	**DISCUSSION**				
14	**Key results**		Summarize key results with reference to study objectives		
15	**Limitations**		Discuss limitations of the study, taking into account the validity of the IV assumptions, other sources of potential bias, and imprecision. Discuss both direction and magnitude of any potential bias and any efforts to address them	10	Lines 295-305: Due to data availability, linkage disequilibrium, and minor allele frequency, these analyses were restricted to GWAS conducted in individuals with European genetic ancestry. We originally sought to compare the effects of the lifelong genetically predicted exposures in MR to RCT studies in shorter windows. We did not achieve this due to the ordinal nature of the diet traits, coupled with the difficulty of identifying RCTs solely testing the food or beverage of interest. We expected that social determinants of health (SDOH) would confound the IVs, yet the PheWAS-TT method mostly identified anthropometric traits or biomarkers. This may be due to the unequal representation of SDOH traits in our PheWAS data, the polygenicity of the traits, or the precision with which anthropometric traits are measured versus SDOH traits and behavioral factors like diet. Nevertheless, it is important to point out that traits that are genetically correlated, such as diet and SDOH, may not be strongly associated with the top diet loci.
16	**Interpretation**				
		a)	Meaning: Give a cautious overall interpretation of results in the context of their limitations and in comparison with other studies	10	Our overall interpretation of the results is provided in lines 306-308: Concern about the credibility of dietary intake-MR is timely, given the growing interest and proliferation of publications in this field. In the absence of biologically informed IVs, PheWAS based approaches cannot be relied on to isolate the direct effect of dietary intake on health.

		b) Mechanism: Discuss underlying biological mechanisms that could drive a potential causal relationship between the investigated exposure and the outcome, and whether the gene-environment equivalence assumption is reasonable. Use causal language carefully, clarifying that IV estimates may provide causal effects only under certain assumptions	9	Oily fish is a rich source of omega-3 fatty acids, which would be a main driver of how oily fish intake reduces triglyceride levels and cardiovascular disease. Alcohol contains ethanol which stresses the liver resulting in higher levels of alanine aminotransferase and liver cirrhosis.<br>Our goal was not to discover new diet-health relationships but rather to address the performance of MR using genome-wide significant instruments coupled with PheWAS filtering approaches when functionally informed variants are unknown.
		c) Clinical relevance: Discuss whether the results have clinical or public policy relevance, and to what extent they inform effect sizes of possible interventions	9	Our goal was not to discover new diet-health relationships but rather to address the performance of MR using genome-wide significant instruments coupled with PheWAS filtering approaches when functionally informed variants are unknown.
17	**Generalizability**	Discuss the generalizability of the study results (a) to other populations, (b) across other exposure periods/timings, and (c) across other levels of exposure	10	The study was performed using exposure data from individuals of European genetic ancestry from the UK Biobank who completed a food frequency questionnaire. Diverse biobanks with rich environmental data including robust measurements of dietary intake should be prioritized.
	**OTHER INFORMATION**			
18	**Funding**	Describe sources of funding and the role of funders in the present study and, if applicable, sources of funding for the databases and original study or studies on which the present study is based	15	The funders had no influence on the results or the decision to publish the results.
19	**Data and data sharing**	Provide the data used to perform all analyses or report where and how the data can be accessed, and reference these sources in the article. Provide the statistical code needed to reproduce the results in the article, or report whether the	7	This study was conducted using publicly available data outlined in Table 1.

		code is publicly accessible and if so, where		
20	**Conflicts of Interest**	All authors should declare all potential conflicts of interest	15	The authors have no relevant financial or non-financial interests to disclose.

This checklist is copyrighted by the Equator Network under the Creative Commons Attribution 3.0 Unported (CC BY 3.0) license.

1. Skrivankova VW, Richmond RC, Woolf BAR, Yarmolinsky J, Davies NM, Swanson SA, et al. Strengthening the Reporting of Observational Studies in Epidemiology using Mendelian Randomization (STROBE-MR) Statement. JAMA. 2021;under review.

2. Skrivankova VW, Richmond RC, Woolf BAR, Davies NM, Swanson SA, VanderWeele TJ, et al. Strengthening the Reporting of Observational Studies in Epidemiology using Mendelian Randomisation (STROBE-MR): Explanation and Elaboration. BMJ. 2021;375:n2233.
